# Management of cervical cancer during the corona virus disease-19 (COVID-19) era

**DOI:** 10.1259/bjr.20200686

**Published:** 2020-11-20

**Authors:** Abhinav Dewan, Swarupa Mitra, Sumeet Aggarwal, Soumitra Barik, Inderjeet Kaur, Preetha Umesh, Rupali Dewan

**Affiliations:** 1 Department of Radiation Oncology, Rajiv Gandhi Cancer Institute and Research Centre, New Delhi, India; 2 Department of Gynecology and Obstetrics, Vardhman Mahavir Medical College and Safdarjung Hospital, New Delhi, India

## Abstract

COVID-19 pandemic has had a catastrophic impact on the society, economy and heath-care system all over the globe with virus showing no signs of losing potency. As the situation appears to worsen, extra burden on other specialities like oncology seems to increase. Specific recommendations are necessary for management of cervical cancer in the current context. All concerned specialities must work together in the best interest of the patient. Attempts should be made at managing cervical cancer while limiting the viral spread among the patients and health-care workers without the loss of opportunity. Surgical intervention for early cervical cancer should be postponed or alternative modalities be considered. In a locally advanced disease, concurrent chemoradiation is the treatment of choice. In addition, the following under mentioned suggestions aim to discuss ways of minimizing infection spread, workload rationalization and providing guidance for management of cervical cancer in the presence of COVID-19 infection.

## Introduction

Corona Virus Disease-19 (COVID-19) was declared a worldwide pandemic by World health organization (WHO) on March 11, 2020. It is known to be caused by SARS-CoV-2 (Severe Acute Respiratory Syndrome Corona Virus 2). COVID-19 disease has resulted in a devastating crisis with an unparalleled impact on economy, medical assets and society worldwide. In view of the rapid host–host transmission, social distancing and lockdowns are the new normal nowadays. Majority of the health-care resources, funding and personnel are being reallocated towards frontline of pandemic control, thereby putting subsequent strain and negative impact on resources available for the remaining specialties including cancer care. Our role as oncologist is to fullfill obligation to our society, cancer patients and follow the WHO motto of attempting to stop, contain, delay and reduce the viral impact whenever possible. Preliminary data indicates increased severity of respiratory illness (4–8 times increased risk) in cancer patients with synchronous COVID-19 disease.^1–3^ Risk of severe and fatal COVID-19 illness is elevated especially in patients with recent history of surgical intervention or cancer chemotherapy.^1^ Curbs have been placed over various elective surgical procedures and patient visits to hospital worldwide; whether this strategy can persist at the cost of standard cancer care raises a few ethical issues. As the incidence and mortality attributed to COVID-19 disease shows a rising trend, the persistent lack of resources and health-care personnel may prevail for an unanticipated long period of time. Variation in health-care facilities and manpower across the globe means that there are no ‘One fit for all’ guidelines available. In the face of challenges posed by the COVID-19 pandemic, we have attempted to review the literature for providing the strategies to optimize clinical practice for cervical cancer patients with maximal safety and efficacy, while alleviating the extra burden on health-care system especially in a developing country like ours.

Cervical cancer is a major public health problem in a developing country like India with an estimated 96,922 new cases reported in the year 2018.^4^ Majority of the cases present in an advanced stage. Delay in timely treatment for such patients may lead to a loss of the window of opportunity and may in turn lead to upstaging of disease with no cure available.

Concerns by patients and treating oncologists have risen with regards to the management protocol of cervical cancer. In the current scenario, delay in diagnosis and treatment is bound to happen. Cervical cancer is considered a ‘Category 1’ disease, *i.e*. rapidly growing tumors with a short volume doubling time in whom prolongation of treatment beyond 2 days should be avoided for 95% of the patients.^5^ Shen et al. noted a significant decrease in 1 year and 5 year survival, if cervical cancer treatment was delayed and delivered >4 months after the initial diagnosis.^6^ Aim should be to provide best possible care for cancer patients keeping in mind the safety of the patients, their families and the health-care workers. Discussion and counselling of patient and attendants regarding the pros and cons of delayed treatment in the current setting including calming their nerves, easing their anxiety etc is essential and is a challenging situation. Various oncological societies have provided recommendations taking into consideration the need to address the cervical cancer and limiting the risk of exposure.^7–11^

Cervical cancer is a category 1 disease and main objective of cervical cancer remains its therapeutic management using either surgical or radiotherapy. Risk–benefit ratio for every therapeutic and diagnostic intervention should be considered and discussed in the gynecological tumor boards and conveyed to the patient and family. Every attempt should be made to reduce the risk of loss of opportunity resulting from delaying treatment or using alternative treatment options. Standard recommendations should be implemented wherever possible during patient management. Prioritization of the patient based upon the intent of treatment, the extent of disease, co-morbidities, patient age, life expectancy and available resources is essential in current COVID-19 context. Diagnostic imaging may be delayed depending upon the impact on patient treatment.

The following considerations have been briefly summarized for management of cervical cancer patients in the present COVID-19 scenario.

### A. Preparedness for Gyne-oncological service

- Promoting principles of social distancing (Stand at a distance of atleast two meters) and adequate hygiene among all patients, relatives, and health personnel.
- Masking and wearing personal protective equipment (PPE)/reusable gowns to be made compulsory for all health-care workers.
- Immunosuppressive patients to be advised to strictly stay at home if possible.
- Spread education regarding prevention, diagnosis and treatment of cervical cancer.
- Paper pamphlets and booklets to be distributed discussing various aspects of COVID-19 infection.
- Consider staggered duty roster for health-care providers to minimize exposure.
- Separate rooms and beds to be designated for quarantining suspected COVID-19 patients.

### B. Outpatient Department (OPD) Visits

- OPD visits limited to patients on treatment, new histologically confirmed patients (with no previous treatment) and patients presenting with acute emergency (Unstable patients with acute abdomen, renal obstruction, post/on treatment complications, severe bleeding).
- Routine follow-up visits of cancer patients to be delayed if possible. European society of medical oncology (ESMO) recommends delay of 2 months after palliative treatment (Advanced/recurrent disease) and 6 months after radical treatment (For Early stage disease) respectively.
- Restricting personnel to absolute minimum that is essential for patient care without impacting the quality of work. Allow work from home if possible.
- Screening of patients entering hospitals at flu corner using thermal scanners.
- Triage of cases with fever, chest symptoms and history of recent travel to international country may be considered for undergoing COVID-19 testing as per the institutional policy.
- Limiting the number of attendants accompanying to one.
- Transition to web-based consultations or telemedicine with proper documentation encouraged for new and follow-up consultations. However, in case of cervical cancer follow-up, a clinical examination is a must and cannot be substituted for by telemedicine. Therefore, it is preferable not to delay follow-up as in other malignancies.
- In case of an emergency or symptoms, telephone or email correspondence should be available to all patients.
- Need to delay any unnecessary interventions especially in asymptomatic patients

### C. Diagnostic Imaging and Routine Work-Up

- Routine imaging including MRI, CT-scan and ultrasound recommended in cases assigned highest priority (Bowel perforation, peritonitis, hydro-uretero-nephrosis, cord/nerve compression, work-up of new patient).
- Proper disinfection of the machine and room to be carried out in case of a suspected COVID-19 case taken up for imaging.
- Attempt to offer home blood collection and portable imaging if possible for high risk patients.
- Medium or low priority is assigned to patients with suspicion of tumor recurrence and for follow-up patients.

### D. Management of Cervical Cancer

Management of cervical cancer should be discussed on case-to-case basis after discussion in the multidisciplinary virtual tumor board meetings comprising of surgical oncologist, radiation oncologist, medical oncologist, pathologist, radiologist and palliative care specialist. Discussion with patient should comprise of the various treatment options, pros and cons of each modality, impact on treatment delay on outcome and increased risk of COVID-19 infection during treatment.

Radiotherapy with or without concurrent chemotherapy is the standard of care for locally advanced cervical cancer and in view of the foreseeable decrease in surgical procedures, should be considered the first line management option for early stage cervical cancer when possible.^12,13^ Extended field radiotherapy is recommended for gross paraaortic disease or electively in case of a) Positive Common Iliac Node and/or b)>3 pelvic nodes.^14^ Decision to delay surgery should be made depending upon the age, co-morbidities of the patient, number of COVID-19 cases in the hospital/nearby areas and facilities available. Patient prioritization is important in deciding on the course of treatment. Surgery should be considered and given highest priority in emergency cases like peritonitis, perforation, post-surgical or radiotherapy complications. ESMO recommends medium priority for: 1) radical hysterectomy with bilateral salpingo-oophrectomy and pelvic lymphnodal dissection for early stage cervical cancer (IA2-IIA) and 2) trachelectomy for Stage IA disease. Lowest priority is assigned to patients supposed to undergo pelvic exenteration, surgery for slow growing central-recurrence, pre-invasive lesions and fistula repair until the end of COVID pandemic if possible. Minimal invasive surgery using laparoscopy is being avoided due to the risks posed by pneumo-peritoneum.^7^ Cases likely to be managed without any adjuvant therapy may be best considered for surgery in the current scenario. Surgical cases likely to suffer from post-op complication, major blood loss and requiring prolonged hospital stay may be considered for an alternative treatment modality as the beds/ICUs and facilities may not be available for routine cancer care and be reserved for COVID-19 patients. In a post-op case with high-risk pathological features (Positive margins/parametrium/lymphnodes), adjuvant therapy is recommended and should be commenced within 4–6 weeks of surgery. For intermediate risk features, adjuvant radiotherapy alone is recommended and may be deferred as it improves disease free survival with no impact on overall survival (OS). COVID-19 testing should be done before all surgical and medical treatments. Informed consent explaining risk of COVID-19 infection during surgery, chemotherapy or radiotherapy should be explained to patient and relatives. A differential diagnosis of COVID-19 should always be considered if patient develops pneumonia during treatment.

Stagewise management of cervical cancer at present should be considered as follows -

- **a. Cervical pre-invasive disease** (Low priority):
- **Low-grade lesions** - Further evaluation/follow-up after 6–12 months High-grade lesions - evaluation to be arranged in within 3 months.
- **High-grade lesions** - evaluation to be arranged in within 3 months.
- **b. Early-stage disease**: standard of care is surgery if feasible in the present scenario. Both surgery and radiotherapy as we known have equivalent local control rates.^14,15^
- **If there is limited access to surgical options -** postpone surgeries considered to be associated with prolonged operative time and increased risk of complications (Radical Hysterectomy) till a resolution of the pandemic or atleast for 6–8 weeks (Medium Priority). In case of prolonged delay (>8 weeks), radical chemoradiotherapy or neoadjuvant chemotherapy may be considered.^6^
  **Microscopic or low-risk (<2 cm and low-risk histology) disease** - Conization or simple trachelectomy with or without sentinel lymphnodal sampling may be considered (Can be postponed for 6–8 weeks) (Medium Priority).
  **Gross visible tumor** – Neo-adjuvant chemotherapy (NACT) considered.^7^
- **c. Locally advanced disease -** (Highest Priority): concurrent chemoradiation is the treatment of choice and should be initiated within 4 weeks of diagnosis (**IB3-IVA**). Hypofractionation is preferred for reducing the hospital visits of patient, thereby minimizing the chances of exposure.
- **d. Metastatic disease (Stage IVB**) - (Highest Priority) – Palliative chemotherapy (Paclitaxel/cisplatin/bevacizumab) or radiotherapy (Cord compression/Brain metastasis).
- **e. Recurrent disease**
- **Local recurrence >12 months after previous chemoradiation** (Highest Priority) - Chemotherapy.
- **Local symptomatic central or para-aortic recurrence** (Medium Priority) – Salvage Radiotherapy.
- **Slow growing central recurrence** (Lowest Priority) **-** Resection/Pelvic exenteration.

## Radiotherapy aspects

### Impact of radiotherapy timing on treatment outcome in cervical cancer

Prolongation of radiotherapy treatment duration negatively impacts local control via tumor repopulation.^16^ Retrospective review assessing the effect of overall treatment duration (external beam radiotherapy/EBRT and brachytherapy) in cervical cancer (Stage I–IV) noted a 1% decrease in local disease control and overall survival for every 1 day delay beyond the median treatment time.^17^ Similarly, various studies have noted an adverse outcome for cervical cancer patients with radiotherapy treatment duration extending beyond 8 weeks (56 days).^17–20^ Tanderup et al.^21^ recently published data of 488 patients of locally advanced cervical cancer treated with radical chemoradiation followed by image-guided brachytherapy. Authors noted an improved 3 year overall survival (OS) with an overall treatment time of less than 7 weeks and recommended an additional 5 Gy radiotherapy dose to compensate for any extension beyond the recommended time.

Total treatment package time of 7–8 weeks is recommended for radical treatment of cervical cancer including chemoradiation and brachytherapy for non-COVID ± PUI/ILI (Person under investigation/Influenzae like illness). Adjuvant EBRT with and without chemotherapy should if possible commence within 4–6 weeks of surgery with an aim of minimal disruption during treatment. Adjuvant therapy can be delayed till 8–12 weeks depending on the modality prescribed (12 weeks for adjuvant EBRT/8 weeks for adjuvant chemoradiation).^22^

## Brachytherapy

Brachytherapy is an essential component of cervical cancer management and should not be delayed if adequate protective gear and health-care personnel are available even in cases of COVID-19 positive or IUI/PUI.^23^ In an infected individual, if a decision to delay brachytherapy boost is taken, then treatment should be resumed 10–14 days after recovery and an additional cumulative brachytherapy dose of 5 Gy per week is necessary if dose constraints for normal tissues are satisfied.^22–24^

Role of post-operative vaginal brachytherapy boost is controversial and may be preferred in cases of adverse risk features like positive margins, lymphovascular invasion, parametrial/vaginal involvement and large/deeply invasive tumor.^25^ It should be avoided after EBRT in the absence of adverse factors especially during this pandemic. Extrapolating from endometrial cancer data, higher dose of 50.4 Gy instead of 45 Gy may suffice instead of a brachytherapy boost. In case of COVID positive patient after 1 or 2 fraction, further sessions may be postponed until 10–14 days after recovery from infection.^22–24^

## Radiotherapy dose fractionation

Use of hypofractionated radiotherapy has seen a rise during the pandemic especially for breast cancer, prostate cancer etc. There are limited data with regards to cervical cancer. Muckaden et al^25^ compared hypofractionated regimen (39 Gy/13#) with the standard fractionated schedule and noted comparable survival in both the treatment arms with Grade 1–2 toxicity. Mendez et al^13^ concluded that a hypofractionated dose of 39–40 Gy at >2.5 Gy per fraction when used in combination with concurrent chemotherapy was associated with a significant treatment response with toxicity comparable to modern treatment series. A Canadian Phase II study titled ‘Hypofractionated External-beam Radiotherapy for Intact Cervical Cancer’ (HEROICC)-trial has started recruitment of the patients and have prescribed a dose of 40 Gy/15# to whole pelvis (BED = 45 Gy/25#) with simultaneous integrated boost to enlarged nodes to a dose of 48 Gy/15# (BED = 57.5 Gy/25#).^13^ Hypofractionated radiotherapy helps to complete the total treatment in a limited period of time, thereby reducing the overall exposure risk and mitigating shortage faced by radiation departments. Altered fractionation may be used if dose constraints as per published data are met.

There is however significant apprehension with regards to the safety and efficacy of the altered fractionation schedules being recommended for locally advanced cervical carcinoma among radiation oncologist all over the world. Apart from few Phase I/II studies, there is no definitive evidence for the same. Use of this alternative strategy for elective para-aortic radiation also needs further evaluation.^13^ Hypofractionated radiotherapy should only be considered with caution and recommended in the context of a institutional review board approved clinical trial and not used as a substitute for conventional fractionation at present.

American brachytherapy society (ABS) has recommended various fractionation schedules for cervical brachytherapy. Shorter fractionation schedules of 2 or 3 sessions should be considered to reduce treatment duration during this pandemic.^26,27^ Rao et al^28^ demonstrated comparable local tumor control with no increase in toxicity and better compliance with three fraction schedule in comparison to the four fraction schedule (8 Gy x 3 fraction *vs* 6 Gy x 4 fraction). Caution is advocated when using two fraction brachytherapy schedules as it may lead to inferior local control.^29,30^ An alternative strategy is to deliver 2–3 fractions 6 h apart per insertion.^31^ In case of interstitial needle implant, a single implantation is preferable with the aim of delivering atleast 4–5 fractions at an interval of 6 h between them.^26,27^ A dose of 5–6 Gy per fraction for two sessions prescribed to 0.5 cm from the surface of the applicator may suffice in cases of vaginal brachytherapy boost after EBRT in the present scenario.

## Brachytherapy and anesthesia recommendations

Brachytherapy is an essential component of radiotherapeutic management of patient with cervical cancer and is often carried out in operation theatre with anesthesia. Analgesics and anesthetic agents are used to provide comfort and relaxation to the patient while performing intracavitary and interstitial brachytherapy. Combination of spinal or epidural anesthesia or local nerve blocks with moderate sedation may provide adequate patient analgesia.^32^ In the present scenario, general endotracheal anesthesia should be avoided as far as possible to reduce chances of exposure of health-care workers to deep respiratory secretions with intubation/extubation. In addition, adequate universal precautions including use of PPE is advisable.

## Image-guided brachytherapy recommendations

In current situation, it is essential to minimize the chance of infection exposure for patients and health-care workers, even due to imaging modalities. Though, MRI helped in improving accuracy of high-risk CTV (HR-CTV) delineation in comparison to a CT scan, Williamson et al.^22^ recommended CT-based brachytherapy planning for local disease restricted to cervix and with limited vaginal involvement (T1b-2a). Authors advised to use MRI sparingly for extracervical disease especially where gray zone delineation was necessary (T2b-T4a). Beriwal et al.^33^ advocated use of MRI-based planning to be restricted to first fraction (especially if there is minimal residual disease). Risk–benefit ratio needs to be assessed for using MRI.

### E. Clinical trials

- Numerous interactions are required between a health-care providers and patients for a clinical trial.
- Number of trials should be limited to accrual of new patients. Only those studies which provide life-saving opportunities over the present standard of care should remain open.
- Patients already on a trial drug should be allowed to continue with the trial treatment.
- In case of a patient becoming COVID positive during the study, such patients should be removed from the study and managed as per institutional protocols.
- Attempts should be made to downsize the research staff currently dealing with the clinical trial.

### F. Educational activities

- Multidisciplinary tumor boards and conferences should be encouraged through web-based telemedicine systems with an aim of preventing delay in patient care.
- Teaching activities for post-graduates and graduates to continue through teleconferencing.
- G. Palliative and hospice services
- Priority of the palliative team is to understand and meet the unmet needs (End-of-life goals, quality of life, pain management, managing emergency situations like uncontrolled vaginal bleeding etc).
- Rapid response teams should be assigned to provide supportive and hospice care to patients at home or hospital in the quickest way possible.
- Teleconsultation is advocated for all patients.
- Keeping family members in loop and frequent counselling is of essence for all palliative care patients.

### H. Preventive services

All routine cancer screening and follow-ups (especially after treatment for early stage disease/pre-invasive disease) to be rescheduled for a later date once pandemic clears out. Proper counselling and reassurance to patients is essential.

Institutional practice varies as far as COVID-19 positive cancer patients are concerned. In general, curative treatment is given a preference and priority over adjuvant treatment. In case of COVID-19 positive or PUI patients, cervical cancer management should be delayed for atleast 2 weeks and patient should be negative on 2 repeat tests done 24 h apart before any further treatment (Surgery/radiotherapy/chemotherapy) is contemplated. If a patient tests positive during radiotherapy treatment, then further management depends upon the institutional policy, patient priority and after taking into consideration the risk–benefit ratio. Immediate radiotherapy treatment interruption with dose adjustment later-on may be considered for such patients. This issue may pose an ethical dilemma for the management, oncologist and treating staff. On one hand, we may be delaying treatment for a curative disease; on the other hand, treatment of such patients may pose a significant risk of spread of infection to other patients as well as health-care workers. In addition, use of a separate linear accelerator, a fully equipped (PPE kit, mask, gloves) radiotherapy team and the complex nature of shifting the infected patient from COVID ward to radiotherapy department is filled with great risks/complexity and may not be a feasible option in all heavy burden departments.^34^ We need to take a balanced decision taking into consideration both aspects.

Given the highest priority assigned to locally advanced cervical cancer, authors advocate concurrent chemoradiation to be considered for asymptomatic COVID-19 patients without treatment breaks. These patients should be treated last on the linear accelerator with the same timeslots maintained for atleast 14 days or until symptom-free for 72 h with 2 negative tests done 24 h apart. Patients should be handled on the treatment machine by a dedicated team of volunteers comprising of radiotherapy technologists, physicians and nurses using infection control protocols and PPE. Weekly reviews by the treating oncologist is a must for such patients using above-mentioned precautions. All rooms should be disinfected at the end of day.^4,35^

## Cancer care after resolution of COVID-19

With resolution of pandemic, routine hospital services may resume. However, balance has to be struck between the new patients and already waiting patients coming to the hospital for treatment. All the depleted facilities, medicine and supplies needs to be replenished before starting any treatment.

## Conclusion

The corona virus disease has provided a unique and serious challenge to the whole world. With a rapid rise in the number of infected cases, oncologists all over the globe should adopt a ‘Do No Harm’ approach. All cancer service providers should try to minimize the risk of infection and take a holistic approach to reconsider the therapeutic and diagnostic indications for all cancer patients. Management decisions should be taken on case-to-case basis as per the prevailing pandemic condition. These suggestions mentioned above may be taken into consideration for management of cervical cancer patients. No guidelines can however act as a substitute for maintaince of adequate hygiene, promoting social distancing and wearing mask at all times.
